# Colorimetric Glucose Biosensor Based on Chitosan Films and Its Application for Glucose Detection in Beverages Using a Smartphone Application

**DOI:** 10.3390/bios14060299

**Published:** 2024-06-07

**Authors:** Anastasia Skonta, Myrto G. Bellou, Theodore E. Matikas, Haralambos Stamatis

**Affiliations:** 1 Laboratory of Biotechnology, Department of Biological Applications and Technologies, University of Ioannina, 45110 Ioannina, Greece; a.skonta@uoi.gr (A.S.); m.bellou@uoi.gr (M.G.B.); 2 Department of Materials Science and Engineering, University of Ioannina, 45110 Ioannina, Greece; matikas@uoi.gr

**Keywords:** chitosan films, glucose oxidase, deep eutectic solvent, glucose, colorimetric biosensors, color recognition application

## Abstract

Nowadays, biosensors are gaining increasing interest in foods’ and beverages’ quality control, owing to their economic production, enhanced sensitivity, specificity, and faster analysis. In particular, colorimetric biosensors can be combined with color recognition applications on smartphones for the detection of analytes, rendering the whole procedure more applicable in everyday life. Herein, chitosan (CS) films were prepared with the deep eutectic solvent (DES) choline chloride/urea/glycerol (ChCl:U:Gly). Glucose oxidase (GOx), a widely utilized enzyme in quality control, was immobilized within CS films through glutaraldehyde (GA), leading to the formation of CS/GOx films. The optimized GOx concentration and DES content were determined for the films. Moreover, the effect of the pH and temperature of the glucose oxidation reaction on the enzymatic activity of GOx was studied. The structure, stability, and specificity of the CS/GOx films as well as the Km values of free and immobilized GOx were also determined. Finally, the analytical performance of the films was studied by using both a spectrophotometer and a color recognition application on a smartphone. The results demonstrated that the films were highly accurate, specific to glucose, and stable when stored at 4 °C for 4 weeks and when reused 10 times, without evident activity loss. Furthermore, the films displayed a good linear response range (0.1–0.8 mM) and a good limit of detection (LOD, 33 μM), thus being appropriate for the estimation of glucose concentration in real samples through a smartphone application.

## 1. Introduction

Quality control in the food industry aims at the evaluation of food characteristics such as flavor and texture before supplying food products to consumers [1]. More specifically, investigating the sugar content of food and beverages is of great importance, since glucose and other sugars are strongly correlated with food quality and human health [2,3,4,5]. So far, conventional analytical methods, such as high-performance liquid chromatography (HPLC) and gas chromatography (GC), have been utilized for glucose detection; however, such methods are costly, time-consuming, and may be destructive [6,7]. Furthermore, conventional analytical methods are laborious and require specialized equipment, which is usually difficult to carry for on-site analyses and is operated by qualified personnel [8,9].

Lately, enzymatic biosensors have emerged as efficient analytical tools to assess food quality [10]. In fact, the analyses are conducted by non-specialized personnel through portable devices rapidly and cost-effectively, as well as sensitively and selectively, thanks to the enzymatic recognition process [9]. Glucose oxidase (GOx or GOD) is the enzyme that has been used for glucose monitoring in blood since 1962 [11], yet it is oftentimes applied for glucose biosensing due to its superior biocatalytic properties [12]. In detail, GOx is a flavoprotein most commonly produced by *Aspergillus niger*, which can catalyze the oxidation of its main substrate, glucose, to D-glucono-δ-lactone and hydrogen peroxide (H_2_O_2_), with the former finally converted to gluconic acid and the latter to water and oxygen [13]. Notably, the produced H_2_O_2_ by GOx can be exploited by horseradish peroxidase (HRP) for the oxidation of a colorimetric substrate, such as 2,2′-azino-bis(3-ethylbenzothiazoline-6-sulfonic acid) (ABTS), 3,3′,5,5′-tetramethylbenzidine (TMB), or o-dianisidine, leading to the formation of a colored product, which can be quantified spectroscopically; this is a useful method for glucose analysis [14,15,16,17].

During the fabrication of biosensors, a critical point is the immobilization of the biocatalysts [18]. Chitosan is a biopolymer widely used for this purpose owing to its biocompatibility, biodegradability, non-toxicity, functional groups for enzyme immobilization, and its ability to create films [19]. Regarding GOx biosensors and their application in food samples, the biocatalyst has been immobilized on modified electrode surfaces in the presence of chitosan either covalently or through entrapment of the enzyme within a chitosan hydrogel [20,21,22]. However, to the best of our knowledge, there is a lack of research on chitosan-based GOx biosensors other than electrochemical ones for glucose determination in food products.

Colorimetric enzymatic sensors comprise an ideal alternative approach for analyte monitoring, as they have a simple and sustainable method that does not require specialized equipment and personnel [23]. The method is based on the detection of color change as a result of the enzymatic reaction, which can be visually perceptible and measured spectroscopically [24]. Recently, color recognition applications available on smartphones and computers were proposed for the colorimetric analysis of glucose simply and on-site by capturing an image of the colored system with a smartphone or an office scanner [25,26,27,28].

In the present work, a novel colorimetric glucose biosensor based on chitosan films was constructed aiming to simplify the procedure of multiple sample analysis without the need for specialized equipment. Firstly, a chitosan solution was mixed with the deep eutectic solvent (DES) choline chloride/urea/glycerol (ChCl:U:Gly). Then, the aforementioned mixture was mixed with GOx and glutaraldehyde, drop-cast on the wells of a well plate, and dried for the covalent immobilization of the enzyme within the formed films. The structural and biocatalytic characteristics of the developed system as well as its analytical performance were studied. Finally, the biosensor was successfully applied to glucose detection in beverages using a smartphone application. To the best of our knowledge, not only is it the first report for enzyme immobilization in chitosan–ChCl:U:Gly films, but also for a colorimetric glucose biosensor based on such films for glucose determination in beverages using a smartphone as a detector and a transducer.

## 2. Materials and Methods

### 2.1. Materials

Low-molecular-weight chitosan (≥75% deacetylated), GOx by *Aspergillus niger*, HRP, glucose, 2,2′-azino-bis(3-ethylbenzothiazoline-6-sulfonic acid (ABTS), and choline chloride (>98%, ChCl) were purchased from Sigma-Aldrich (Saint Louis, MO, USA). Urea (>99%, U) was obtained from Fluka (Charlotte, NC, USA). Glycerol (0.5% max water, Gly) and glutaraldehyde (25%, GA) were bought from Fisher Scientific (Waltham, MA, USA).

### 2.2. Preparation of the DES ChCl:U:Gly

The DES ChCl:U:Gly was prepared by mixing appropriate amounts of choline chloride, urea, and glycerol at a molar ratio of 1:1:1 [29]. The mixture was incubated at 80 °C and underwent an intermediate vortex until a clear solution was obtained.

### 2.3. Preparation of Chitosan Films

A 1.5% *w*/*v* chitosan solution was prepared by dissolving 1.5 g of chitosan in 100 mL of a 1% *v*/*v* aqueous acetic acid solution and incubating it overnight at 70 °C under constant stirring [29]. The solution was subsequently centrifuged at 9500 rpm for 15 min and the insoluble particles were discarded. The chitosan solution was then mixed with the ChCl:U:Gly solution in the concentration range of 0–5.68% *v*/*v* and incubated under magnetic stirring for 1 h at room temperature for the complete homogenization of the mixture. For the immobilization of GOx, the protocol of Yao et al. [30] was followed with some adaptations, which was further combined with the concept of enzyme immobilization on chitosan-coated well plates as in Elchinger et al. [31]. Firstly, the stock solutions of GOx (0.25–2 mg/mL) and GA (2.5% *v*/*v*) were prepared by dissolving both in 50 mM phosphate buffer, pH 7.0. Appropriate volumes of the GOx and GA stock solutions were mixed with an appropriate volume of the chitosan–DES solution to reach the final concentrations of 0.025–0.2 mg/mL, 0.05% *v*/*v*, and 0–5% *v*/*v* for GOx, GA, and DES, respectively. For the formation of the films, 250 μL of the solution was drop-cast on 96-well plates and dried at 30 °C. After, the formed films (CS/GOx) were rinsed with 50 mM phosphate buffer, pH 7.0, thrice. For the formation of blank CS films (without GOx), the same procedure was followed, but a buffer solution was added instead of the GOx-containing one.

### 2.4. Structural Characterization of the Films

The structural characterization of the films was carried out using attenuated total reflection (ATR). The films that were used for this experiment were neat CS films (0% *v*/*v* DES) and CS-DES films (3% *v*/*v* DES) without GA and GOx (CS and CS-DES, respectively), with GA and without GOx (CS-GA and CS-DES-GA, respectively), and with GA and GOx (CS-GA-GOx and CS-DES-GA-GOx, respectively). The films were prepared by drop-casting 2.5 mL of the final film-forming solution to a 12-well plate with final concentrations of GOx and GA equal to 0.1 mg/mL and 0.05% *v*/*v*, respectively. The samples were placed at the ATR PRO ONE accessory of a Jasco FT/IR 4700 spectrometer (JASCO, Tokyo, Japan) and a total of 64 scans at a resolution of 2 cm^−1^ were recorded in the range of 400–4000 cm^−1^.

### 2.5. Optimization of CS/GOx Film Preparation

The assay for the measurement of the enzymatic activity of GOx is based on the combination of two reactions [14,15] (Equations (1) and (2)). The first reaction is the catalysis of glucose oxidation by GOx, which leads to the production of gluconic acid and H_2_O_2_. In the second reaction, HRP catalyzes the oxidation of ABTS owing to the presence of H_2_O_2_, resulting in the formation of ABTS^+^, a blue-green solution with absorbance at 405 nm.

(1) $$Glucose+O_{2}\;\overset{GOx}{\to }Gluconic\;acid+H_{2}O_{2}$$

(2) $$ABTS+H_{2}O_{2}\;\overset{HRP}{\to }\;{ABTS}^{+}+H_{2}O$$

For the selection of the optimized GOx concentration and DES content, the CS/GOx films with GOx concentrations and DES content ranging between 0.025 and 0.2 mg/mL and 0 and 5% *v*/*v*, respectively, were studied. The CS/GOx and CS (blank) films were incubated with 200 μL of the reaction solution, which consisted of glucose (0.25 mM), ABTS (0.5 mM), and HRP (10 μg/mL), for 5 min at pH 7.0 and 30 °C. The solutions were then transferred to the clean wells of an ELISA well plate and the absorbance of the solutions was read at 405 nm. The Beer–Lambert law was applied for the calculation of the produced ABTS^+^ concentration. One unit (1 U) of enzymatic activity is equal to the amount of the enzyme that catalyzes the production of 1 nmol of ABTS^+^ per minute under the above-described reaction conditions. The experiment was conducted in triplicate.

### 2.6. Effect of pH and Temperature on the Enzymatic Activity of GOx

For the determination of the effect of the reaction pH on the enzymatic activity of free GOx, the reaction solution contained glucose (0.25 mM), ABTS (0.5 mM), HRP (10 μg/mL), and GOx (2.5 μg/mL) in a total volume of 200 μL, all prepared in 50 mM citrate phosphate buffer pH 4.0 and 5.0; in 50 mM phosphate buffer pH 6.0, 7.0, and 8.0; and in 50 mM Tris/HCl buffer pH 9.0. The reaction solution was incubated at 30 °C for 5 min and its absorbance was measured at 405 nm in 1 min intervals in an ELISA reader. The effect of the reaction temperature on the enzymatic activity of free GOx was studied as indicated previously, except for the pH, which was fixed at 7.0, and the temperature, which varied between 30 and 50 °C. The relative activity was calculated for the different conditions by defining the highest enzymatic activity as 100%.

The effect of the pH and temperature on the enzymatic activity of immobilized GOx was investigated as described in Section 2.5. More specifically, the CS/GOx and CS (blank) films were incubated with 200 μL of the reaction solution, namely glucose (0.25 mM), ABTS (0.5 mM), and HRP (10 μg/mL). The films were incubated for 5 min at the appropriate pH and temperature and the absorbance was measured at 405 nm at the end of the incubation period after transferring the solutions to clean wells of an ELISA well plate. The experiment was conducted in triplicate.

### 2.7. Kinetic Study of GOx

The kinetic study of free GOx was performed by mixing glucose (0.25–200 mM), ABTS (2 mM), HRP (20 μg/mL), and GOx (0.5 μg/mL) in a total volume of 200 μL in 50 mM phosphate buffer, pH 7.0. Each mixture was incubated at 30 °C for 5 min and its absorbance was measured at 405 nm at regular time intervals.

The kinetic study of immobilized GOx was conducted by incubating the films with a mixture of glucose (0.025–4 mM), ABTS (2 mM), and HRP (20 μg/mL) in a total volume of 200 μL in 50 mM phosphate buffer, pH 7.0. The films were incubated at 30 °C for 5 min and their absorbance was finally measured at 405 nm. A Michaelis–Menten plot was created for both forms of GOx and the Km values were obtained. The kinetic constants were calculated using nonlinear regression analysis (EnzFitter, Biosoft, UK). The experiment was conducted in triplicate.

### 2.8. Precision Evaluation and Storage Stability of Immobilized Gox

The precision of the CS/GOx films was investigated in terms of reusability (repeatability) and reproducibility. The reusability of immobilized GOx was evaluated for 10 reaction cycles by measuring the enzymatic activity of the biocatalytic system after 10 successive incubations with the reaction solution consisting of glucose (0.25 mM), ABTS (0.5 mM), and HRP (10 μg/mL) at pH 7.0 and 30 °C for 5 min. The remaining activity was calculated for each cycle concerning the initial enzymatic activity, which was defined as 100%. After each reuse cycle, the films were washed with the buffer solution thrice.

The reproducibility of the CS/GOx films was studied by incubating six different films with the above-mentioned reaction solution under the same conditions. The results were expressed as relative standard deviation (RSD).

The storage stability of the immobilized enzyme was studied during 4 weeks of storage at 4 °C and room temperature (RT). Briefly, the initial enzymatic activity of immobilized GOx was determined as described previously and was defined as 100%. Afterwards, the films were stored at 4 °C and RT under dry conditions. Every week, a different triplet of CS/GOx and blank films was used to determine the remaining enzymatic activity. All experiments were conducted in triplicate.

### 2.9. Analytical Performance of the Biosensor through UV/Vis Spectroscopy

The efficiency of the constructed biosensor was assessed in terms of its linear range, the limit of detection (LOD), the limit of quantification (LOQ), and specificity towards glucose. The linear response range was determined by the plot of glucose concentration versus the absorbance. The LOD and LOQ values were calculated using Equations (3) and (4) [32]:

(3) $$LOD=\frac{3.3\times \sigma }{S},$$

(4) $$LOQ=\frac{10\times \sigma }{S},$$

where *σ* is the standard deviation of the response of the blank samples and *S* is the slope of the calibration curve.

The specificity test of the immobilized GOx was performed by checking the ability of various sugars to be used as alternative substrates. In essence, except for glucose, fructose, maltose, and sucrose were used at the concentration of 1 mM. The reaction solution of the sugar (1 mM), ABTS (2 mM), and HRP (20 μg/mL) was transferred to the CS/GOx films, and after 5 min of incubation at 30 °C the absorbance was measured as described in the previous paragraphs (Section 2.5). All experiments were conducted in triplicate.

### 2.10. Analytical Performance of the Biosensor through a Color Recognition Application

The linear range, LOD, and LOQ of the CS/GOx films were determined through a color recognition application as well. In detail, photographs of the colored reacted solutions in Section 2.7 and Section 2.9 were captured after their removal from the film-containing wells and their transport to clean ones. For this purpose, a smartphone (Xiaomi Redmi 9T, Xiaomi Communications Co., Ltd., Beijing, China) was used as a camera and an analysis tool. The distance between the phone and the samples was fixed at 20 cm, the color of the background was white, and direct illumination was avoided to capture the images under the same light conditions. The freely available application “Color Grab” was downloaded on the device and utilized for the color analysis among the different samples. The Color Grab application allows the user to detect colors from an image simply and quickly, providing information on the most common color models. Moreover, real-time color measurement is feasible, which is a useful feature of the application since color can be picked in real-time through the phone’s camera without the need to have captured a photograph beforehand. Here, after image acquisition, the color was picked from the images at the center of each sample and far from glaring spots, and various color parameters were obtained. More specifically, the “Value” parameter of the Hue, Saturation, Value (HSV) color space, which gives insight into the brightness of the colors and can receive values between 0 and 100% [33], was selected for the quantification of the results. The linear range of glucose was determined by plotting the glucose concentrations with their corresponding values. The LOD and LOQ values were calculated by Equations (3) and (4). The experiment was conducted in triplicate.

### 2.11. Determination of the Glucose Content in Real Samples through a Color Recognition Application

The glucose content in beverages was determined through a commercially available biosensor (Contour next, ASCENSIA Diabetes Care) and the developed CS/GOx films. Here, the real samples were apple juice, pineapple juice, mixed fruit juice (with 9 different fruits), a lemon–lime soft drink with zero sugar, and soda water. Firstly, glucose solutions of known concentrations were prepared and used to create a calibration curve, which correlated the known glucose concentrations with the concentrations detected by the commercial biosensor. Then, the apple juice, pineapple juice, and mixed fruit juice were diluted appropriately with 50 mM phosphate buffer, pH 7.0, so their glucose content fell into the measuring range of the commercial biosensor (10–600 mg/dL or 0.56–33.3 mM). However, this step was not necessary for the lemon–lime soft drink with zero sugar and soda water. First, the glucose content of the beverages was calculated by using the commercial biosensor and the calibration curve of glucose. In order to calculate the glucose content using the CS/GOx films, at least three CS/GOx films as well as blank ones were simultaneously incubated for 5 min at 30 °C and pH 7.0, with the following reaction solutions: (i) glucose (0.1 mM), (ii) glucose (0.8 mM), and (iii) real sample (5 μL from 10× diluted solution of apple juice or mixed fruit juice, 15 μL from 20× diluted solution of pineapple juice, or 15 μL from the undiluted lemon–lime soft drink and soda water). The above reaction solutions also contained ABTS (2 mM) and HRP (20 μg/mL) in a total volume of 200 μL. At the end of the incubation period, the solutions were transferred to the clean wells of an ELISA well plate to terminate the reaction and a photograph of all the solutions was taken under the same light conditions. The selection of the glucose concentrations, 0.1 and 0.8 mM, was because each represents the lowest and highest value of the linear response range towards glucose in the case of the color recognition application, as will be discussed further in the Results section. The obtained images were imported into the Color Grab application and the color was picked at the center of each solution. The value parameters of 0.1 and 0.8 mM glucose solutions were used each time to create a 2-point calibration plot and its equation was used to calculate the unknown concentrations of the beverages. The experiment was conducted in triplicate.

### 2.12. Spiking Study in a Real Sample through a Color Recognition Application

The accuracy of the proposed CS/GOx biosensing films was evaluated with the spiking study. For this purpose, 4 CS/GOx films along with blank films were incubated at pH 7.0 for 5 min at 30 °C with glucose (0.1 mM and 0.8 mM), pineapple juice (15 μL from 20× diluted solution), and pineapple juice with a known added concentration of glucose (0.2 mM). Accordingly, the same procedure was followed for the lemon–lime soft drink with the difference that, instead of juice, 15 μL from the undiluted lemon–lime soft drink was used as the sample and the same known concentration of glucose (0.2 mM) was added. These reaction solutions also contained ABTS (2 mM) and HRP (20 μg/mL) in a total volume of 200 μL. The procedure for image acquisition and analysis was the same as that in Section 2.10. The recovery rate of spiked glucose was calculated using Equation (5). The experiment was conducted in triplicate.

(5) $$R(\%)=\frac{spiked\;sample\;result-unspiked\;sample\;result}{known\;spike\;added\;concentration}\times 100\%$$

## 3. Results

**Figure 1:**
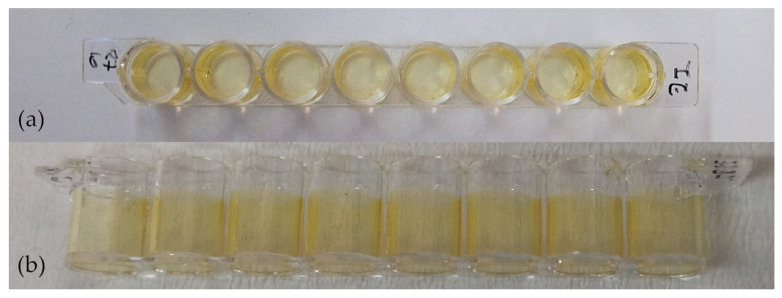
CS/GOx films prepared in the wells of an 8-well ELISA strip (yellowish color): (**a**) top view of the films; (**b**) side view of the films.

In the present study, a colorimetric glucose biosensor was developed by implementing GOx within CS films (Figure 1). The DES ChCl:U:Gly was implemented in the film preparation in order to improve its mechanical and physicochemical properties, acting as a plasticizer, based on another report where the employment of U-based DES was studied [34]. GA was added not only to enhance the stability of the biocatalyst, but also to exploit the possible crosslinking properties of U and GA [35]. The concentration of glucose in the test samples could be determined by incubating the CS/GOx films with the test solution, capturing photographs of the reacted solutions with a smartphone, and processing the data with a smartphone application. To the best of our knowledge, this is the first time that a chitosan smartphone-based GOx colorimetric biosensor has been developed and applied in food glucose quantification.

### 3.1. Structural Characterization of the Films

**Figure 2:**
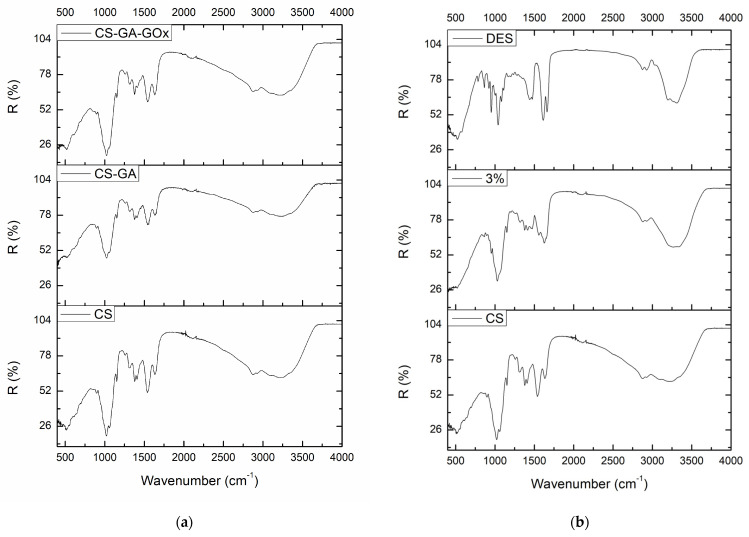
ATR spectra of (**a**) CS neat films, CS-GA and CS-GA-GOx films; (**b**) CS neat films, CS films with 3% *v*/*v* DES, and DES.

The structural properties of the CS films were characterized using ATR spectroscopy. The spectra of neat chitosan films (0% *v*/*v* DES) with and without GA and GOx as well as their DES-containing counterparts (3% *v*/*v* DES) were obtained and are presented in Figure 2. With regard to the neat chitosan film (Figure 2a), the polymer’s characteristic bands are observed, as described elsewhere [29]. The characteristic bands of the saccharide structure appear in the range 800–1150 cm^−1^ [30], referring to the C-C and C-O bonds [36], with the band at 1152 cm^−1^ corresponding to the C-O-C of the saccharide ring [37]. The bands at 1320 cm^−1^ (Amide III), 1378 cm^−1^, and 1410 cm^−1^ correspond to C-N stretching of N-acetyl residues [38], acetamide groups, and carboxylate groups [36], respectively. Also, the bands appearing at 1538 cm^−1^ (Amide II) and 1634 cm^−1^ (Amide I) are assigned to the bending of the primary amine’s N-H bond and to the stretching vibration of the amide group’s C=O bond [36,39], while the broadband at 3000–3600 cm^−1^ is attributed to the N-H and O-H stretching vibrations [36].

Focusing on the impact that GA and GOx had on the chemical structure of chitosan, no significant changes were detected, which is in accordance with the results of another study examining the interaction of GOx immobilized in a chitosan film [30]. Probably, the concentration of GA that was used (0.05% *v*/*v*) was not enough to cause major changes in the spectrum, contrary to the results of other studies [37,40,41] where a higher concentration of GA was used and an increase in the intensity of a band around 1650–1660 cm^−1^ (C=N bond) occurred. However, herein, a small shift of the band at 1634 cm^−1^ to 1639 cm^−1^ is observed in the CS-GA film that could probably be assigned to the formation of the C=N bond between the amine groups of CS and carbonyl groups of GA, overlapping with the band that is related to the vibration of the C=O bond. Analogous small shifts and overlaps are also reported elsewhere [39], indicating the crosslinking of GA with CS. Furthermore, when GOx was added, this shift disappeared and the band reappeared at 1633 cm^−1^ with slightly increased intensity, in comparison to the spectra of CS and CS-GA films. This increase in the intensity of the Amide I band and the possible overlapping of the peaks of C=O and C=N vibrations could be attributed to the presence of GOx [30,42].

When DES is incorporated in the CS film, significant changes are observed in the IR spectrum, which can be attributed to the presence of the DES (Figure 2b). Nevertheless, the main bands of the CS structure are still detected, indicating that the polymeric core still exists. It is noteworthy that a significant shift in the band at 1538 cm^−1^ to 1557 cm^−1^ occurs and changes at the 3000–3600 cm^−1^ region are observed, as similarly reported in another study [34]; these are possibly attributed to the interaction between CS and DES. No considerable changes were observed among CS-DES, CS-DES-GA, and CS-DES-GA-GOx films (Figure S1), and thus, although the DES’s existence can be noticed in the DES-containing films, it seems that its presence obstructs the detection of significant differences between the unmodified and the GA-GOx modified CS films.

### 3.2. Optimization of CS/GOx Film Preparation

**Figure 3:**
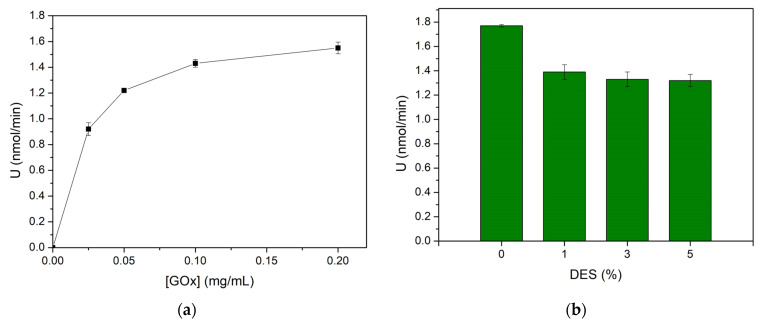
Effect of (**a**) GOx concentration (0.025–0.2 mg/mL); (**b**) DES content (0–5%) in the enzymatic activity (U) of the immobilized GOx.

The preparation of the CS/GOx films was optimized in terms of GOx concentration and DES content. Firstly, the effect of the initial GOx concentration was studied in the range of 0.025–0.2 mg/mL in films containing 1% *v*/*v* DES. The enzymatic activity of the CS/GOx films was determined over this range of GOx concentrations and the results are depicted in Figure 3a. An increase in the GOx concentration from 0.025 mg/mL to 0.1 mg/mL led to a pronounced increase in the enzymatic activity. A possible explanation is that by increasing GOx concentrations more enzymatic molecules are immobilized and catalytically available. Nevertheless, a less intense increase was noted in the range of 0.1–0.2 mg/mL. This could be because at a certain point, the immobilization carrier becomes gradually saturated and fewer GOx molecules are efficiently attached. The same trend was observed in the work of Susanto et al. regarding the effect of the initial GOx concentration on the binding capacity of the enzyme [40]. Considering the enzymatic activity of the biocatalyst and the cost of the process, the GOx concentration was selected to be equal to 0.1 mg/mL.

DESs are green solvents that are formed by mixing one or more hydrogen bond donors (e.g., U, Gly) and a hydrogen bond acceptor (e.g., ChCl) [29]. So far, various choline chloride-based DESs have been used for the fabrication of chitosan films, due to their plasticizing effect [34,43]. Here, the ChCl:U:Gly DES was investigated as a plasticizer. Various amounts of the DES solution were mixed with the chitosan solution, leading to final concentrations in the range of 1–5% *v*/*v*, and the enzymatic activity of GOx was measured in each case (Figure 3b). As can be seen in Figure 3b, the highest enzymatic activity is observed at 0% DES, whereas about a 21–25% reduction is obtained in the presence of the DES. However, the 3% *v*/*v* content of the DES was selected as the optimum, since a better plasticizing effect was realized in that case, enabling the reusability of the films. Similarly, the addition of the DES ChCl:U in a CS solution for the formation of CS films resulted in more flexible films [34].

### 3.3. Optimization of Reaction Conditions

**Figure 4:**
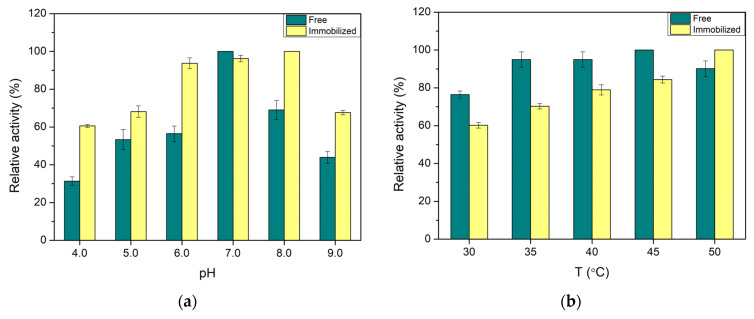
Effect of (**a**) pH; (**b**) temperature (T) on the activity of free and immobilized GOx in CS/GOx films.

The effect of the reaction pH and temperature on GOx activity was also investigated to optimize the bioprocess conditions and, thus, the biosensor efficiency. For the pH study, buffer solutions with pH values in the range of 4.0–9.0 were studied for their effect on the enzymatic activity of free and immobilized GOx. As shown in Figure 4a, the free biocatalyst displays its highest activity at pH 7.0, with GOx activities for the rest of the tested pH values being significantly lower. The immobilization of GOx resulted in enhanced relative activity in the pH range of 6.0–8.0, while the relative activity for the other pH values was significantly higher than that of free GOx. These results are in agreement with other reports where it is claimed that enzyme immobilization offers a stabilizing effect over enzyme activity [44,45,46].

The influence of the reaction temperature on GOx activity was examined in the range of 30–50 °C (Figure 4b). Free GOx exhibited its lowest relative activity at 30 °C; however, at 35–50 °C its activity remained practically the same. On the contrary, the relative activity of immobilized GOx increased drastically with increasing reaction temperatures from 30 to 50 °C. The same trend was observed for free and immobilized GOx in the study of Haskell et al.; the relative activity of free GOx increased with increasing the temperature from 30 to 35 °C, and the values of relative activity were very close at 35–45 °C, but at 50 °C declined intensively, while the relative activity of the immobilized enzyme increased with increasing the temperature from 30 to 45 °C, but at higher temperatures it started to decrease [44]. Moreover, Ateş and Içli observed the same trend for immobilized GOx as in the present work; the relative activity increased with increasing temperatures and finally started to decline at 55 °C [47]. This behavior could be the result of the creation of a favored transition state of the enzyme–substrate complex at temperatures higher than 30 °C [48]. Considering the prerequisites of a sustainable bioanalytical method and in order to ensure high enough activity and neutral conditions, the reaction of glucose oxidation was decided to be conducted at pH 7.0 and 30 °C.

### 3.4. Kinetic Studies of Free and Immobilized GOx

**Figure 5:**
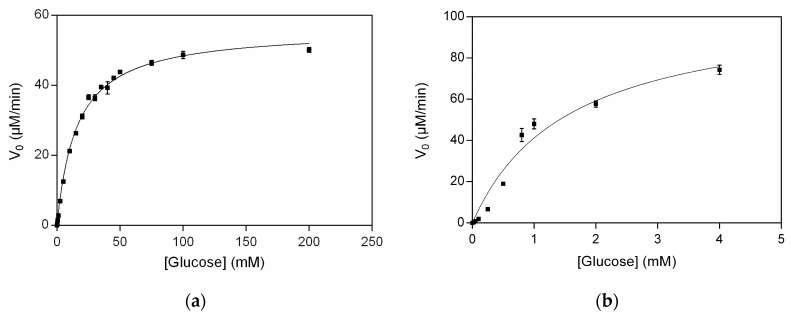
Michaelis–Menten plot of (**a**) free GOx; (**b**) immobilized GOx in CS/GOx films.

The Km value, a kinetic parameter indicative of the affinity of the enzyme towards its substrate, was measured by incubating the free GOx and CS/GOx films with various glucose concentrations. The Michaelis–Menten plot of the free and immobilized GOx is presented in Figure 5. The Km value of free GOx was calculated to be 15.77 ± 0.60 mM, while that of immobilized GOx was 1.56 ± 0.24 mM. Therefore, the immobilization of GOx increased its affinity towards glucose by almost 10 times, possibly due to an improved arrangement of GOx molecules and a better accessibility of the substrate to the active site [15]. The Km value of free GOx has been reported to have various values in the range of 9–22 mM [15,48,49,50,51], which could be related to the existing differences between the assays. It is worth noting that the Km values of immobilized GOx in the present study and in the study of Yao et al., on which the fabrication of our film was based, were very close (1.56 mM and 2 mM, respectively) [30]. Thus, the immobilization of GOx in the CS/GOx films exerted a positive impact on its catalytic activity.

### 3.5. Precision Evaluation and Storage Stability of Immobilized GOx

**Figure 6:**
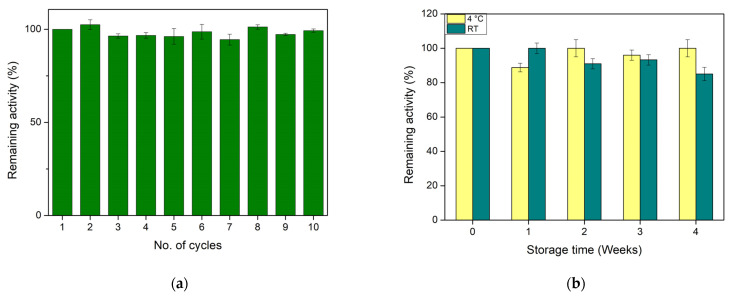
Evaluation of (**a**) reusability; (**b**) storage stability of immobilized GOx in the CS/GOx films at 4 °C and room temperature (RT).

Some desired characteristics of biosensors are precision (reusability and reproducibility) and storage stability for long periods [42]. Therefore, the optimized biocatalytic system of CS/GOx films was studied regarding these features. More specifically, the reusability of CS/GOx films was measured for 10 consecutive cycles of glucose oxidation at 30 °C. The results are depicted in Figure 6a with the remaining activity of the first cycle being defined as 100%. Interestingly, during the 10th reaction cycle, the system practically retained all its initial activity (99 ± 1%). Similarly, the immobilization of GOx in magnetic cellulose nanocrystals led to the maintenance of 100% of its initial activity during the 10th reaction cycle [15]. However, in comparison to similar work, where GOx was immobilized on polyphenol-coated multi-well plates, the biocatalyst retained about 30% of its initial activity during the fifth cycle of operation [50]. The enhanced reusability of GOx in our study could be attributed to the covalent linkage of GOx molecules within the chitosan matrix through GA, which protected the enzyme molecules from detachment [52].

The reproducibility of CS/GOx films, that is, the similarity of the obtained results by different films under the same reaction conditions, was evaluated through the RSD value [42]. Here, the reproducibility of CS/GOx films was found to be good, since the RSD value of six films was equal to 3.27%, which is acceptable as it is lower than 4% [22]. Correspondingly, in the study of Ang et al. the reproducibility was 9.46% for low-molecular-weight chitosan films with covalently immobilized GOx [42], while in the study of Artigues et al. the RSD was 2.5% for a biocatalytic system composed of immobilized GOx using a chitosan hydrogel onto highly ordered titanium dioxide nanotube arrays [22].

In addition, the CS/GOx films were tested for their storage stability. The remaining activities of CS/GOx films, which were stored at 4 °C and RT during 4 weeks of storage, are presented in Figure 6b with the remaining activity of the biocatalyst being defined as 100% for the initial activity measurement (week 0). The CS/GOx films were proven to be more stable when stored at 4 °C rather than RT, since in the former case GOx did not lose any of its initial activity after 4 weeks of storage, while in the latter case, it lost only 15% of its initial activity. These results further support the fact that immobilization plays a significant role in the stabilization of enzymes. Notably, GOx immobilized in a similar chitosan network matrix retained 90% and 62% of its initial activity after 2 weeks and 2 months of storage at 4 °C under dry conditions, respectively [30]. In the study of Yee et al., the remaining activity of GOx after the first week of storage at 4 °C dropped to 70% and it remained constant for the following 3 weeks [15]. The storage stability of GOx at RT was investigated in the study of Bai et al., where immobilized GOx retained approximately 43% of its original activity after 21 days of storage [51]. Nevertheless, encapsulated GOx in a paper-based glucose sensor demonstrated high stability for up to 6 weeks of storage at RT [53]. All in all, the proposed CS/GOx films could be considered as more sustainable than the above-mentioned systems, as CS/GOx films combine both reusability and storage stability, not only at 4 °C but also at RT. Thus, the developed films can be utilized with high precision and stability in biosensing applications, features that increase the economic viability of the system.

### 3.6. Specificity Study of Immobilized GOx

**Figure 7:**
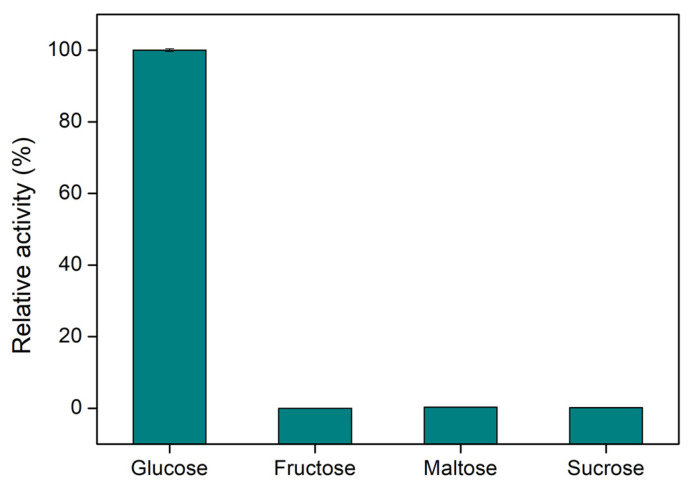
Specificity study of GOx in CS/GOx films towards glucose, fructose, maltose, and sucrose.

Specificity is a very important characteristic of biosensors, since in order to be applicable in complex samples, they should respond only to the analyte of interest. The specificity of CS/GOx films was tested by incubating distinct films with solutions of glucose, fructose, maltose, and sucrose at concentrations of 1 mM. Figure 7 depicts the results of the specificity study, expressed in units of relative activity (%), with the relative activity of GOx towards glucose being defined as 100%. As can be seen from the graph, the developed CS/GOx films were highly specific to glucose, as they displayed zero activity when fructose, maltose, and sucrose were used instead of glucose. Hence, CS/GOx films can be utilized for the specific detection of glucose.

### 3.7. Analytical Performance of the Biosensor through UV/Vis Spectroscopy

**Figure 8:**
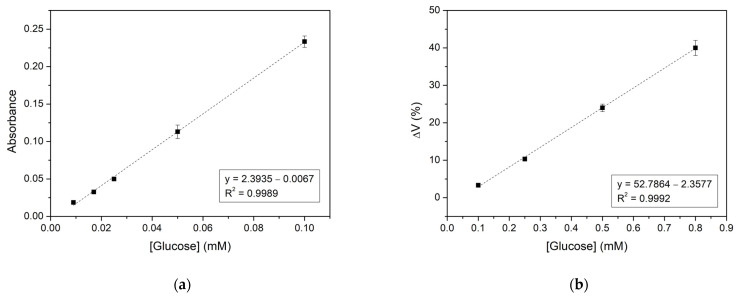
Calibration curve of glucose using CS/GOx films determined by (**a**) UV/Vis; (**b**) color recognition application.

Subsequently, the analytical performance of CS/GOx films was evaluated towards glucose detection. For this purpose, the films were incubated with reaction solutions containing glucose in the range of 0.009–4 mM. Right after the incubation period, the absorbance of the solutions was measured spectrophotometrically, and the glucose calibration curve was created (Figure 8a). The linear response range towards glucose was determined by the calibration curve and was found to be 0.009–0.1 mM. The LOD and LOQ were also calculated and were equal to 2 and 7 μM, respectively. A comparison of the results obtained in this work and in similar recent works of glucose colorimetric sensors based on UV/Vis measurements is presented in Table 1. More specifically, the lower limit of the glucose linear range and LOD in the present study are lower than those referred to in the literature (Table 1), indicating that the developed CS/GOx films are capable of detecting lower glucose concentrations. On the contrary, the upper limit of the linear range is not as high as in other studies, which could be addressed by properly diluting the samples of higher concentrations so their absorbance values fall into the linear range.

### 3.8. Analytical Performance of the CS/GOx Films through a Color Recognition Application

**Figure 9:**
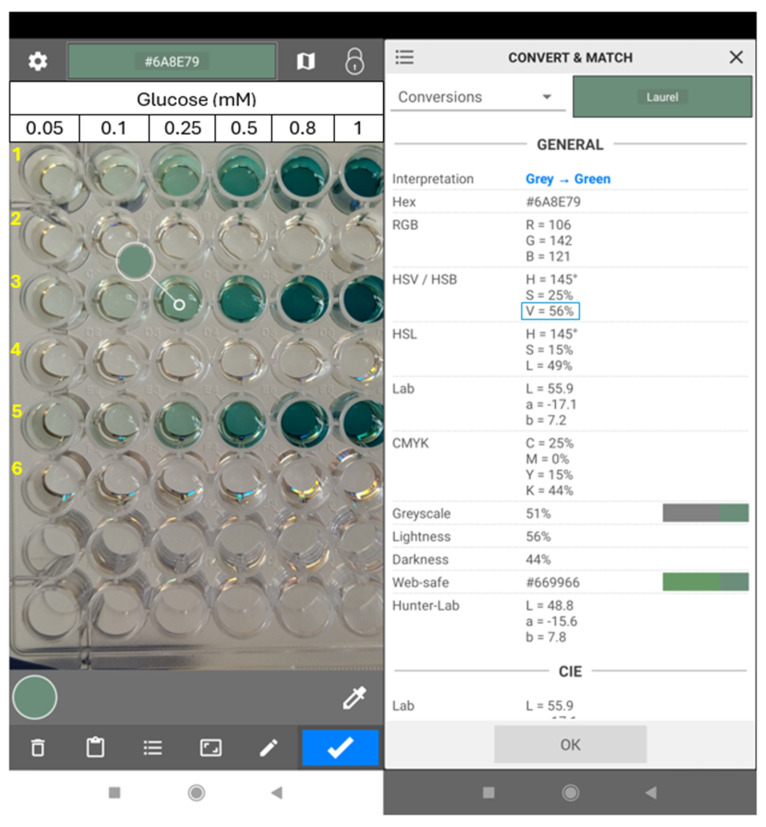
Photograph of the user interface of the Color Grab application. Color pick of the reacted solutions (**left**) and report of the color parameters (**right**). The “Value” parameter is highlighted with a blue frame. Lines 1, 3, and 5: glucose solutions 0.05–1 mM after incubation with CS/GOx films; lines 2, 4, and 6: respective blank solutions.

The analytical performance of the CS/GOx films was also studied using a color recognition application on a smartphone as an attempt to simplify the analysis procedure and render it more applicable for everyday use. So far, various applications and color parameters have been used to calculate analyte concentrations through colorimetric biosensors [55]. Here, the reaction solutions, whose absorbance was previously measured spectrophotometrically, were photographed and the color parameters were extracted for the selected areas of the images in the Color Grab application (Figure 9). Among the different obtained parameters, the Value parameter from the HSV color space was found to be more suitable for the analysis in the present study. Thus, glucose concentration was plotted versus the value parameter and a calibration curve was obtained as well (Figure 8b). In that case, the linear response range towards glucose was 0.1–0.8 mM, the LOD was 33 μM, and the LOQ was 99 μM. Comparing these results with those previously obtained by a spectrophotometer, it can be inferred that the LOD, the LOQ, and the lower and upper limits of the linear range are higher when using the color recognition application. The same trend was observed for the LOD based on the spectrophotometric analysis and the smartphone application on poly(aniline-co-anthranilic acid) (ANI-co-AA) composite films, where either GOx or a combination of GOx-HRP was immobilized [26] as well as on a cerium oxide-based hypoxanthine biosensor [56]. It should be noted that several dilutions of the sample may be required to obtain values in the linear range. Moreover, the LOD in our work and others’ with smartphone-based glucose analysis do not seem to differentiate to a great extent (Table 2) [53,54], and in some cases, it appears to be lower in our approach [26,57]. Thus, the proposed colorimetric CS/GOx films could be applied to the smartphone-based determination of glucose content in real samples.

### 3.9. Determination of Glucose Concentration in Real Samples Using the CS/GOx Films and the Smartphone Color Recognition Application

Finally, the CS/GOx films were utilized to determine the glucose concentration in apple juice, pineapple juice, mixed fruit juice, lemon–lime soft drink (0% sugar), and soda water. To this aim, different CS/GOx films were incubated with diluted or undiluted samples of the beverages containing HRP and ABTS as well as two films that were incubated with glucose solutions corresponding to the lower and upper limit of the linear response range. The inclusion of the lower and upper limits was essential, since all the samples were photographed simultaneously, and a two-point calibration curve was created to calculate the glucose content of the beverages. It is important to mention that the photographs were taken in different light conditions, in order to evaluate the effect of light on the final results regarding the determination of glucose concentration in the solutions of unknown concentration. Interestingly, when all the solutions were photographed under the same conditions, the calculation of the glucose concentration of a solution with the smartphone application was practically the same among the different light conditions. So, an advantage of this setup is the ability to use it in real conditions, without the need for specific dark boxes or lights. 

Using the color recognition application, the initial concentration of glucose in apple juice, pineapple juice, mixed fruit juice, lemon–lime soft drink, and soda water was calculated and was found to be equal to 152.9, 93.4, 213.6, <0.1, and <0.1 mM, respectively (Table 3). The results were verified by a commercial glucose biosensor. In detail, the glucose concentration in apple juice, pineapple juice, mixed fruit juice, lemon–lime soft drink, and soda water was determined with the commercial biosensor as 172.6, 108.0, 173.6, <0.6, and <0.6 mM, respectively. Nevertheless, it should be noted that the commercial biosensor itself may have an error in the measurements, since the accepted deviation for glucose concentrations < 100 mg/dL (<5.55 mM) is ± 15 mg/dL (± 0.83 mM), whereas for glucose concentrations ≥ 100 mg/dL (5.55 mM) it is ± 15%. Thus, the developed CS/GOx films could be used effectively to estimate the glucose content in real samples by using the color recognition application.

The accuracy of the CS/GOx films was studied last. For this purpose, a sample from pineapple juice was diluted and spiked with a known glucose concentration (0.2 mM). Afterward, its concentration was calculated with the smartphone application, so that the recovery rate could be determined. The glucose concentration of the unspiked pineapple juice sample in the diluted sample was 0.35 ± 0.01 mM, while in the spiked sample was 0.52 mM. The experiment was also conducted for the lemon–lime soft drink, which did not contain the target analyte. The glucose concentration of the unspiked lemon–lime soft drink sample was 0 mM, while that in the spiked sample was 0.21 ± 0.02 mM. Therefore, the recovery rate was equal to 86 ± 4% for the pineapple juice and 105 ± 8% for the lemon–lime soft drink, which appertain to the acceptable range of recovery values (>80%) [58], proving the high accuracy of the CS/GOx films.

## 4. Conclusions

In the present study, CS/GOx films were developed with cheap raw materials and proposed as colorimetric biosensors. Firstly, CS-DES-containing films were prepared with GOx being immobilized within the films. Afterwards, the preparation of the CS/GOx films was optimized regarding the GOx concentration and DES content, since the presence of the ChCl:U:Gly DES led to films with improved plasticity. Moreover, the pH value of 7.0 and the temperature of 30 °C were selected for the reaction of glucose oxidation. The system of CS/GOx films was proven to demonstrate higher affinity towards glucose than the free form of GOx as well as to be stable for over 10 uses and 4 weeks of storage at 4 °C and at RT. The analytical performance of the films was also studied and the linear response range towards glucose, the LOD, and the LOQ were determined both spectrophotometrically (0.009–0.1 mM, 2 μM, and 7 μM, respectively) and by a color recognition application available on smartphones (0.1–0.8 mM, 33 μM, and 99 μM, respectively). Finally, the films were successfully applied to glucose determination in real samples (apple juice, pineapple juice, mixed fruit juice, lemon–lime soft drink, and soda water) by using the color recognition application, and the results were verified by a commercial glucose biosensor. Overall, the films were found to be ideal for colorimetric glucose detection, since they exhibited high precision, accuracy and stability, specificity to glucose, and LODs comparable to others referred to similar works. Hence, the proposed biosensor configuration could simplify the procedure of glucose analysis and lower its cost and the required time, since the analysis can be performed simultaneously for multiple samples by using a smartphone without the need for specialized equipment. In future experiments, natural dyes that are responsive to pH changes caused by gluconic acid production could be incorporated into the films, thus eliminating the need for utilization of HRP and ABTS and reducing the overall cost of the biosensor. Furthermore, additional experiments could be performed in biological fluids to investigate whether the developed biosensor could be used in healthcare applications.

## Figures and Tables

**Table 1:** Comparison of the analytical properties (linear range, LOD, LOQ and real sample) of the CS/GOx films and other glucose colorimetric sensors in the literature based on UV/Vis measurements.

Immobilization Support	Linear Range (mM)	LOD (μM)	LOQ (μM)	Real Sample	Ref.
Magnetic cellulose nanocrystals	0.25–2.5	83	-	-	[15]
Polyamide microparticles	0.01–3.0	53.7 (in urine)	-	Human urine	[54]
Electrospun blended chitosan–poly(vinyl alcohol) nanofibers	2.7–13.8	-	-	-	[17]
Polyphenol-coated multi-well plates	0.0244–6.25	-	-	Rat plasma	[50]
Poly(ANI-co-AA) composite film	0.025–0.2	95 (GOx) 27.7 (GOx-HRP)	-	Red and white wine, pear juice, fresh pomegranate juice, and pharmaceutical product	[26]
CS films	0.009–0.1	2	7	Apple juice, pineapple juice, mixed fruit juice, lemon–lime soft drink, and soda water	This work

**Table 2:** Comparison of the analytical properties (linear range, LOD, LOQ, and color recognition application) of the CS/GOx films and other glucose colorimetric sensors in the literature based on color recognition applications on smartphones.

Immobilization Support	Linear Range (mM)	LOD (μM)	LOQ (μM)	Recognition Application	Ref.
Filter paper	2.78–29.97	1370	-	Self-developed	[57]
Cu_3_(PO_4_)_2_⋅3H_2_O hybrid microflowers	0.001–1	0.3	-	Not specified	[25]
Poly(ANI-co-AA) composite film	0.025–0.2	109 (GOx) 49.4 (GOx-HRP)	-	ColorLab	[26]
Pluronic-based nanocarrier	-	22	-	Color Grab	[53]
Polyamide microparticles	0.01–3.00	30.7 (in urine)	-	Photoshop	[54]
CS films	0.1–0.8	33	99	Color Grab	This work

**Table 3:** Glucose concentration of apple juice, pineapple juice, mixed fruit juice, lemon–lime soft drink, and soda water, determined by the CS/GOx films and the commercial biosensor.

	[Glucose] (mM)	
Sample	CS/GOx Films	Commercial Biosensor
Apple juice	152.9 ± 3.4	172.6
Pineapple juice	93.4 ± 1.7	108.0
Mixed fruit juice	213.6 ± 3.4	173.6
Lemon–lime soft drink (0% sugar)	<0.1	<0.6
Soda water	<0.1	<0.6

## Data Availability

Not applicable.

## Supplementary Materials

The following supporting information can be downloaded at https://www.mdpi.com/article/10.3390/bios14060299/s1, Figure S1: ATR spectra of CS-DES films, CS-DES-GA and CS-DES-GA-GOx films. The DES content of the CS-DES films was 3% *v*/*v*.
