# Supplementary material for: Colorimetric Glucose Biosensor Based on Chitosan Films and Its Application for Glucose Detection in Beverages Using a Smartphone Application
Source: Biosensors (Basel). 2024 Jun 7;14(6):299. doi: 10.3390/bios14060299 (PMC11201573; doi:10.3390/bios14060299)
Supplement: Supplementary file 1 [file biosensors-14-00299-s001.zip › biosensors-3024239-supplementary.pdf]

Supplementary Materials

Colorimetric Glucose Biosensor Based on Chitosan Films and Its Application for Glucose Detection in Beverages Using a Smartphone Application

Anastasia Skonta [1], Myrto G. Bellou [1], Theodore E. Matikas [2] and Haralambos Stamatis [1,*]

[1] Laboratory of Biotechnology, Department of Biological Applications and Technologies, University of Ioannina, 45110 Ioannina, Greece; a.skonta@uoi.gr (A.S.); m.bellou@uoi.gr (M.G.B.)
[2] Department of Materials Science and Engineering, University of Ioannina, 45110 Ioannina, Greece; matikas@uoi.gr
* Correspondence: hstamati@uoi.gr

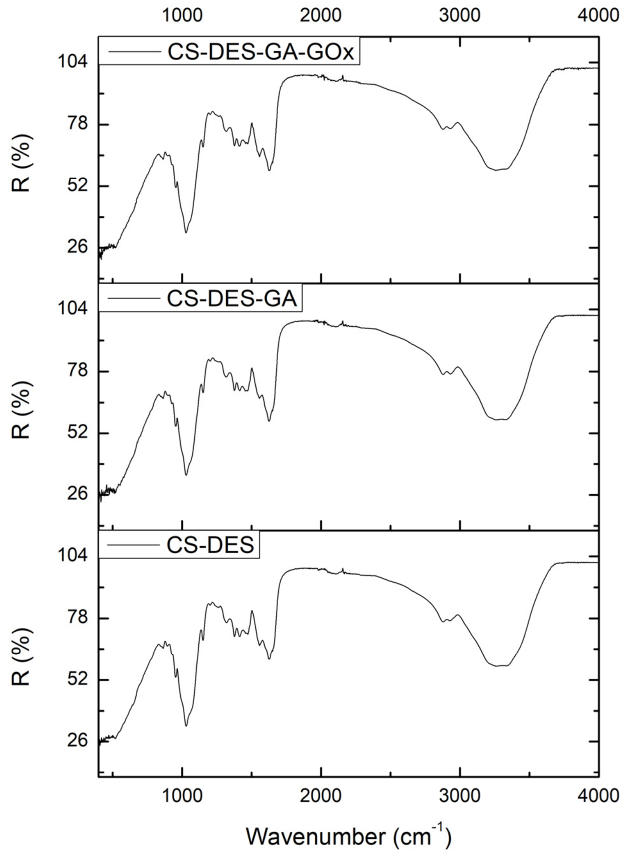

Figure S1. ATR spectra of CS-DES films, CS–DES–GA and CS–DES–GA–GOx films. The DES content of the CS–DES films was 3% *v*/*v*.
